# Extra-Linguistic Cognitive Functions Involved in the Token Test: Results from a Cohort of Non-Aphasic Stroke Patients with Right Hemisphere Lesion

**DOI:** 10.3390/bs12120494

**Published:** 2022-12-03

**Authors:** Benedetta Basagni, Silvia Pancani, Leonardo Pellicciari, Paola Gemignani, Emilia Salvadori, Sara Marignani, Antonello Grippo, Bahia Hakiki, Andrea Mannini, Donata Bardi, Ilaria Pellegrini, Maria Pia Viggiano, Fabio Giovannelli, Claudio Macchi, Francesca Cecchi

**Affiliations:** 1 IRCCS Fondazione Don Carlo Gnocchi, Via di Scandicci 269, 50143 Florence, Italy; 2 IRCCS Fondazione Don Carlo Gnocchi, Via Fontevivo 127, 19125 La Spezia, Italy; 3 SODc Neurofisiopatologia, Dipartimento Neuromuscolo-Scheletrico e degli Organi di Senso, AOU Careggi, Largo Brambilla 3, 50134 Florence, Italy; 4 Section of Psychology-Department of Neuroscience, Psychology, Drug Research and Child’s Health (NEUROFARBA), University of Florence, Viale Pieraccini 6, 50139 Florence, Italy; 5 Department of Experimental and Clinical Medicine, University of Florence, Largo Brambilla 3, 50134 Florence, Italy

**Keywords:** Token Test, right hemisphere stroke, memory, comprehension

## Abstract

Background: The Token Test (TT) is widely used to examine comprehension disorders in aphasic patients, but abilities other than language may affect a patient’s performance. This study aims to explore the correlation between the TT subtest performances and the performances in extra-linguistic cognitive areas in a cohort of patients from the Intensive Rehabilitation Post-Stroke (RIPS) study with a first, right hemisphere stroke and without aphasia, prospectively enrolled at admission to intensive inpatient post-acute rehabilitation. Methods: The patients were administered the TT (50-item version), the forward and backward digit span (DST), and the Montreal Cognitive Assessment (MoCA). Spearman’s partial correlations adjusted by age were used to evaluate the association between the number of errors in the TT and the other tests’ corrected scores. Results: Of the 37 patients enrolled in this study, 29.7% made 3–11 errors on the TT, 27.0% more than 11 errors, mostly in parts IV and V. The forward and backward digit span scores showed correlations with errors in part V of the TT (r = −0.408, *p* = 0.013; r = −0.307, *p* = 0.027). The errors in part IV of the TT presented a correlation with a forward digit span too (r = −0.394, *p* = 0.017). With respect to MoCA domains, executive functioning, and orientation were related to the TT part V errors (r = −0.468, *p* = 0.007; r = −0.499, *p* = 0.003). The orientation also correlated with the TT part III (r = −0.504, *p* = 0.002). Conclusion: Our findings show that the TT performances in patients with right hemisphere stroke and without aphasia are related to impairments in auditory–verbal span/auditory working memory mostly for TT scores on subpart V as measured by the DST and to executive function and orientation, as measured by the MoCA subtests.

## 1. Introduction

The Token Test (TT) [1,2,3] is widely used test to assess auditory comprehension in persons with developmental and acquired disorders affecting language, where the patient is verbally required to provide a gestural response (pointing to or moving plastic tokens) in response to a verbal command. Most versions include 20 tokens with different forms (i.e., rectangles and circles), sizes (i.e., large and small), and colors (i.e., red, blue, green, yellow, and white). During the test, the examiner provides a set of increasingly difficult commands, requiring token identification and/or manipulation. Since its first publication in the early sixties, the use of the TT in clinical practice has rapidly spread and the tool has been translated into more than 40 languages [4].

Over the past years, several versions have been proposed, that differ in the number of items, type of stimuli, type of population, and scoring modalities; a digital version is also available [5] and, recently, a version for mobile phones has been proposed [6].

Although the TT has been specifically developed to assess auditory language comprehension, its performance involves other cognitive processes, as it often happens with neuropsychological tests. Such processes include verbal short-term memory, working memory, and inhibitory control, that is the ability to ignore distracting and competing lexical information [7]. This is consistent with the hypothesis of the role of verbal short-term memory [8,9], along with executive functions [10,11], in sentence comprehension. Thus, TT versions with several items presenting increasing length and syntactic complexity of the orders are more likely to involve verbal span and working memory.

TT is included in widespread batteries for Aphasia, such as the Aachener Aphasia Test, (AAT) [12], originally developed in German and later translated into many other languages, including Italian [13]. The Italian version is composed of five parts. In parts I–IV, the patient is verbally required to identify at first one and then more tokens, identified by a progressively increasing number of features. In part V, the examiner requires the patient to manipulate tokens, by providing verbal commands with a complex syntactic structure. Since the last sections of this TT may require quite a long time, some authors pointed out that the overall score may not truly represent verbal comprehension alone and, therefore, suggested examining the performances for parts I to IV and for part V separately [14,15]. Indeed, using such long versions of TTs could be misleading, considering the requirements over working memory abilities, which are frequently reduced in individuals with aphasia [16,17].

Several studies have attempted to assess which functions, other than language abilities, may influence TT performance [18,19,20,21,22,23,24,25] but the results are somewhat conflicting. The crucial role of verbal and non-verbal memory components in TT performance was first reported by Lesser et al. [18]. Namely, a significant correlation between the last section of the TT and the non-verbal measures of the visual and gestural sequencing span was observed, supporting the hypothesis that the TT may overload the auditory–verbal sequencing span in patients with left brain damage [18]. More recently, [23], the relationships between verbal working memory, sentence comprehension, and severity of impairment in aphasic patients with a history of left hemisphere cerebrovascular accidents have been explored. The listening and reading versions of the computerized revised TT that assesses the sentence comprehension and the sentence span task as a measure of the working memory were used: the patients with low compared to high memory capacity, compared to those with high working memory scores, showed a significantly worse performance in the computerized revised TT, particularly in the subtests with syntactically more complex structures [23]. These findings support the view that the working memory’s influence on verbal comprehension is mainly evident when working the memory capacity is taxed by task demands [26,27,28].

Other evidence, however, does not support this relationship. For example, in a study conducted on a large sample of veterans with acquired brain lesions, Koenigs et al. [29] observed that patients with damage to the inferior frontal and posterior temporal regions were impaired either on digit span or on language tests. However, regression analyses revealed that the digit span performance was significantly associated only with the tests assessing language production (i.e., Boston Naming Test and WAIS Vocabulary subtest), whereas no association was found with the TT.

Cognitive control has also been linked to receptive language (10) (24). In these last papers the role of inhibitory control, tested by the Stroop test, was evaluated on auditory comprehension in patients with Wernicke’s aphasia. A significant negative correlation was observed; namely, the greater the Stroop interference effect, the lower the TT performance.

Based on these premises, we aimed to investigate which other components besides language comprehension may affect TT performance. To pursue this goal, we studied TT performance in stroke patients with right hemisphere lesion (RHL). Although patients with RHL may experience communicative–linguistic difficulties (in particular, communication disorders such as turn-taking, argument management, and using non-literal language [30,31] and contextually appropriate language and understanding figurative meaning and the social rules of conversation [32,33]), RHL does not seem to involve the lexical–sematic circuits related to the pure linguistic comprehension.

Within the context of a large prospective study on predictors of rehabilitation outcomes in patients addressing intensive inpatient post-stroke rehabilitation, the Intensive Rehabilitation Post-Stroke (RIPS) study [34], we verified the association between each of the five TT sub-tests and the ability to maintain and manipulate information in working memory (as tested by forward and backward digit spans, respectively) and with the cognitive areas investigated by Montreal Cognitive Assessment, following Aiello et al. [35] grouping, in a sample of non-aphasic, right hemisphere stroke patients.

## 2. Methods

### 2.1. Participants

The data analyzed in this study come from a multicenter observational prospective study, investigating the predictors of functional outcomes at discharge from inpatient post-stroke rehabilitation, the RIPS study [34]. This study involved four intensive rehabilitation units of Fondazione Don Carlo Gnocchi (Firenze, La Spezia, Massa, and Fivizzano). The study protocol was registered a priori on ClinicalTrials.gov (Registration number: NCT03968627) and approved by each center’s local ethics committees (Florence: 14513; La Spezia: 294/2019; Massa and Fivizzano: 68013/2019). The study was conducted following the principles of the Declaration of Helsinki.

All the people admitted to either of the four rehabilitation units from December 2019 to December 2020 were systematically assessed for eligibility and recruited upon signing informed consent. The participants meeting the following inclusion criteria were included in this study: (1) adults (aged ≥ 18 years), (2) first-ever ischemic or hemorrhagic right stroke, diagnosed clinically and with brain imaging, (3) 30 days or less from the index event, (4) first-ever admission to the rehabilitation center for the considered condition, (5) Italian mother tongue, and (6) Scoring 0 at *The National Institutes of Health Stroke Scale (NIHSS)* [36] aphasia item. The patients were excluded from the study if they presented a severe acquired brain injury (sABI), due to a hemorrhagic or ischemic stroke causing disorders of consciousness states and critical clinical care conditions, and if they presented a diagnosis of cognitive deterioration before the stroke.

For this analysis, we used data from right-handed patients, with a right hemisphere lesion, for whom the TT [13], together with the digit span (forward and backward) [37], were available for this analysis. The exclusion criteria were the presence of aphasia at the onset evaluation by item n. 9 of the NHISS (NHISS item n. 9 score > 0), hemineglect at the Oxford Cognitive Screen (OCS), Italian version [38], and sensory/motor deficits that did not allow test administration and data collection. We also used the preliminary test of the TT to exclude patients unable to perform the test for lack of collaboration.

### 2.2. Evaluation Tools

The NIHSS [36] is a widely used tool to assess the severity of acute stroke. It is an 11-item test assessing the main domains of stroke-related disability: level of consciousness, gaze anomalies, visual field restriction, facial palsy, motor arm, and leg limitations, limb ataxia, sensory deficits, aphasia, dysarthria, and extinction and inattention. Each subtest variably scores between 0 and 4 or less and the total score of NIHSS is obtained by their sum. Given a maximum total score of 42, a score of 0 represents a normal function, whereas higher scores indicate more severe degrees of impairment. Item n.9 assesses the presence and severity of aphasia.

The Montreal Cognitive Assessment (MoCA) test [39] is a cognitive screening, composed of brief cognitive tasks that assess visuospatial/executive skills, attention, naming, language, abstraction, delayed recall, and orientation. Administration time is approximately 10 min. The total score ranges from 0 to 30 points. Higher scores reflect better performance. The recent Italian version by Aiello and coauthors [35] provides a grouping of cognitive functions in six areas:

1. *Executive Functioning:* this part is composed of three tests: (a) an alternation task adapted from the trail-making B task, (b) phonemic fluency, and (c) verbal abstraction task;
2. *Attention*: this part is composed of three tests: (a) serial backward subtraction, (b) letter detection by tapping, and (c) forward/backward digit span task;
3. *Language*: this part is composed of two tests: (a) naming of three images of low-familiarity animals and (b) repetition of two syntactically complex sentences;
4. *Visuospatial*: this part is composed of two tests: (a) three-dimension cube copy and (b) clock drawing task;
5. *Orientation*: this part is composed of one task in which the patient is asked to answer specific questions over time and place;
6. *Memory*: this part is composed of a single memory test composed of delayed recall of five nouns after approximately five minutes from a learning trial.

According to Italian normative data, the score should be corrected for age and education. The scores included in this study were raw scores, adjusted according to the correction grids provided in the normative data [35].

Token Test (TT) [13]: The version used in this study is composed of a preliminary test, in which 5 square tokens of different colors are placed horizontally on the table. The patient must indicate the token corresponding to a certain color. This test allows excluding associated cognitive, linguistic, visual, or visual exploratory difficulties, which may cause the test to be unreliable. The test is composed of 5 parts of 10 items each. In the first part, the patient is presented with 5 circles and 5 rectangles of 5 different colors. The patient is asked to identify the target with two features: color and shape (e.g., “Touch the yellow circle”). In the second part, the choice is between 10 circles (i.e., 5 small and 5 big) and 10 rectangles (i.e., 5 small and 5 big) and the patient is asked to identify the target with three features (e.g., “Touch the small white rectangle”). In the third part, the choice is between 5 big circles and 5 big rectangles of 5 different colors. The patient is required to identify two targets, with two features each (e.g., “Touch the red circle and the green rectangle”). In the fourth part, the choice is between 10 circles (i.e., 5 small and 5 big) and 10 rectangles (i.e., 5 small and 5 big) and the patient has to identify two targets, with three features each (e.g., “Touch the white large circle and the small green rectangle”). Lastly, the fifth part is supposed to detect impairment in grammatical processing; the choice is between 5 big circles and 5 big rectangles of 5 different colors and the patient has to perform some simple actions with the tokens (e.g., “after picking up the green rectangle, touch the white circle”). The total score is global and represented by the totality of errors (range 0–50 errors). According to Italian normative data, no effect of age or education has been found. For this reason, the raw score does not need to be corrected. The participant’s performance is scored as “normal” when the global score lies within the highest 95% of the population, corresponding to less than 3 errors, while it is scored as “pathological” when the global score falls within the lowest 5%, corresponding to more than 11 errors, and as “borderline” if it falls between these two values [13].

The forward and backward digit span (FDS) test [37]: FDS mainly measures the verbal short-term memory storage capacity, defined as the system that allows for the temporary storage of information. The patients are presented with sequences of digits (verbal) items that they have to reproduce immediately after the presentation in the same temporal order. The length of the sequence is progressively increased and the span is the longest sequence correctly reproduced (range 0–8 points). The Backward Digit Span (BDS) test mainly measures the verbal working memory, defined as the system that allows for retaining information for a brief period while performing mental operations on that information. The patients are required to reproduce the presented sequences in reverse order. The length of the sequence is progressively increased and the span is the longest sequence correctly reproduced (range 0–8 points). Each raw score is corrected for age and education, following the reference normative data [37]. According to Baddeley’s working memory model [40], the FDS test is regarded as a measure of verbal short-term memory, while the BDS test is regarded as a measure of verbal working memory.

The NIHSS was assessed upon admission to the rehabilitation ward, the other neuropsychological tests were performed within a week of admission.

### 2.3. Statistical Analysis

The scores corrected for age, sex, and education were used for all the following analyses. Firstly, the Shapiro–Wilk test was performed for all continuous variables to determine whether the data were normally distributed. The variables were then summarized as mean and standard deviation (SD) or median and interquartile range (IQR), according to the test results. The categorical data were presented as frequencies with percentages. To investigate whether there was an association between the number of errors performed on the TT and neuropsychological tests adjusted partial correlations were conducted. To take into account the effect of age on TT performance, age was introduced in the analyses as a confounding factor. The absolute value of the correlation coefficient (r) represents a very strong correlation if above 0.80, a strong correlation if between 0.60 and 0.79, a moderate correlation if between 0.40 and 0.59, a weak correlation if between 0.20 and 0.39, and a very weak correlation if below 0.19 [41].

The differences between different lesion locations (grouped into three main categories: subcortical, lobar, or other) were assessed using the Kruskal–Wallis test. The post hoc Bonferroni correction for multiple comparisons was applied. In all the analyses, a *p*-value < 0.05 was considered statistically significant. The analyses were carried out using the SPSS 20.0 software package (SPSS, Inc., Chicago, IL, USA).

## 3. Results

Out of the 278 stroke patients who were eligible for the RIPS study, 235 (85%) signed the informed consent and were enrolled. Of these, 37 met the inclusion criteria and were included in this analysis. The participants had a mean age of 73.5 years (SD: 13.9 years, range 41–94 years). Most of the participants were male (*n* = 23, 62.2%). Twenty-two participants (59.5%) had an ischemic stroke, while 15 (40.5%) had a hemorrhagic stroke. Most of the participants (*n* = 16) had a lobar lesion (43.2%); 37.8% (*n* = 14), presented a lesion in subcortical structures. There were seven (18.9%) participants with any other lesion locations. The specifics on the lesion site for individual subjects are reported in the Supplementary Materials. The demographic and clinical characteristics of the sample are presented in Table 1.

The FDS corrected score obtained by participants ranged between 3.5 and 7.9 (mean value of 5.7). The lower scores were obtained in the BDS test where values ranged from 1.8 to 7.1 (mean value 4.0). A performance lower than 5% of the normative sample was observed in 10.8% of the participants for FDS (score not exceeding 4.26) and 8.1% of the participants for BDS (score not exceeding 2.65). The MoCA mean corrected score obtained by participants was 19.5 (SD: 3.8); the higher score was recorded in the *orientation area* (median score: 5, IQR: 1.7), while the lower score was recorded in the *memory area* (median score 1.6, IQR: 1.9).

The neuropsychological test scores obtained by the participants are summarized in Table 2.

The median number of errors in the TT was 3, ranging from 0 to 25. In particular, 43.2% of participants made less than 3 errors, 29.7% made from 3 to 11 errors, and 27.0% made more than 11 errors, mostly concentrated in parts IV and V. The TT errors by age tertiles are reported in the Supplementary Materials (Table S1). The Spearman’s correlation analysis showed a significant negative correlation between the number of errors performed on the TT and the digit span scores. In particular, the corrected FDS scores had a weak–moderate correlation with the errors recorded in the TT subparts IV and V, with the strongest correlation observed with subpart V (r = −0.408, *p* = 0.0013). No significant correlation was found between FDS and subparts I–III (r = −0.064 *p* = 0.712; r = −0.210 *p* = 0.219; and r = −0.219 *p* = 0.200, respectively). The corrected BDS scores correlated only with the TT subpart V, (r = −0.371, *p* = 0.026). Subparts I–IV showed no significant correlation with BDS (r = −0.181 *p* = 0.290; r = −0.066 *p* = 0.702; r = −0.296 *p* = 0.080, and r = 0.010 *p* = 0.954, respectively (Table 3).

The significant moderate negative correlations were found between the score obtained in the *executive functioning* and *orientation* domain of the MoCA test and TT errors (subparts V) (r = −0.468, *p* = 0.007 and r = −0.499, *p* = 0.003). A correlation was also found between orientation and TT subpart III (r = −0.504, *p* = 0.002) (Table 4).

**Figure 1:**
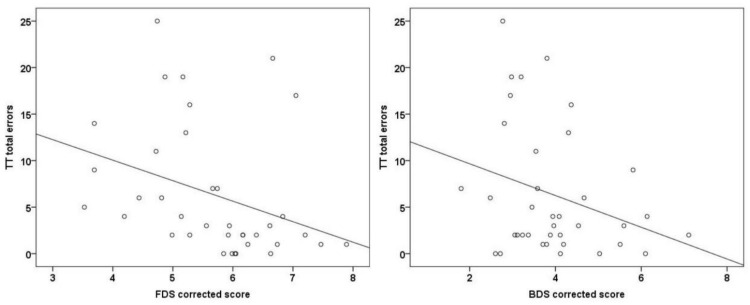
Distribution of Token Test (TT) total errors according to the corrected score obtained in the Forward (FDS) and Backward (BDS) Digit Span test. The solid black line represents the line of best fit.

The TT total error distributions according to the corrected score obtained in FDS and BDS tests are presented in Figure 1.

When the association between the number of TT total errors and the lesion location was investigated, no significant differences were observed (*p* = 0.485) in the number of errors according to the lesion site. Similarly, no significant difference was found in FDS (*p* = 0.629) and BDS (*p* = 0.568) corrected scores between participants with subcortical, lobar, or other lesion locations.

## 4. Discussion

This study aims to investigate which other cognitive domains, besides auditory–verbal comprehension, may affect performance in a TT. To perform this, we conducted a correlation study to explore the association between the TT performance and extra-linguistic factors (i.e., the ability to maintain and manipulate information in working memory and the global cognitive status) in a group of thirty-seven patients with right hemispheric stroke and without aphasia; these were prospectively recruited within a multicentric observational study. Although it is not possible to completely exclude an influence of the right hemispheric lesions on the verbal production necessary for the execution of the neuropsychological tests, by the choice of these inclusion/exclusion criteria, we aimed at excluding a significant linguistic component to the TT performance, while taking into consideration other non-verbal cognitive aspects that could be affected by a stroke.

In order to exclude aphasic patients, we decided to select right-handed patients with unilateral right brain regions. To prevent the risk of including persons with inverted cortical representation, the aphasia NIHSS score was used to exclude patients with aphasia following right hemisphere stroke. This choice was aimed at focusing on potential pathological performances in the TT, most likely unrelated to verbal comprehension problems.

Our sample included patients with stroke, which occurred within 30 days before the recruitment, of either ischemic or hemorrhagic etiology, by a proportion of either in line with the literature [42,43]. The site of the brain injury was evenly distributed between the subcortical and lobar, with a mild global index of severity at NIHSS. Concerning global cognitive functioning, the average MoCA score corresponded to moderate impairment. In our sample, almost 60% of the patients made two or more errors on the TT. This first result suggests that, in patients with stroke, TT performance can be compromised also by aspects other than aphasia. Some of our patients presented short-term and working memory impairment (a performance lower than 5% of the normative sample, in 10.8% for FDS and 8.1% for BDS). A significant negative correlation between the number of errors performed in the TT subpart IV and V and the digit span scores was found. In particular, the errors performed in subparts IV and V showed a weak–moderate correlation with the FDS score, while BDS correlated only with TT subpart V. Interestingly, only part V correlates both with FDS and BDS. Part V is constituted of long orders, with complex syntactic constructions that need a high span and working memory load. Additionally, part IV is composed of long orders, although without syntax complexity. Coherently, part IV correlates with the FDS, while unexpectedly part IV does not correlate with BDS.

In our analysis, we did not observe any difference between the forward and backward span, used as measures of short-term memory and working memory, respectively. This was rather unexpected, since working memory, namely the capacity to simultaneously process verbal information, should be theoretically more related to complex sentence decoding, than the simple maintenance of linguistic information over a short period (verbal span) [44]. However, unlike previous works (e.g., Gajardo-Vidal et al., [45] that included patients tested >3 months and <10 years after their stroke), our patients were in the immediate post-acute phase, within one month from the index event. This may have contributed to the lack of finding a significant difference between the simple short-term information storing and the more complex elaboration ability, since the presence of confounding factors such as attention impairment are more typical of the acute phase.

Among the cognitive models of verbal short-term memory, the phonological loop integrated into the working memory model proposed by Baddeley [40] is probably the most influential. The phonological loop is a limited capacity subsystem involved in the temporary store of verbal information controlled by a supervisory system (i.e., the central executive). According to this model, the verbal span represents the passive temporary maintenance of information, whereas working memory refers to both the maintenance and manipulation of information. Although recent studies have further investigated this concept, and using neuroimaging techniques [46,47,48], the original model is still acknowledged within the scientific community. The auditory short-term memory capacity was found to depend on the structural integrity of a posterior region of the superior temporal gyrus and sulcus [49], while a system involving the auditory cortex and projections from higher-order areas, including the hippocampus and frontal cortex, is considered to be crucial for actively maintaining sounds in memory [50]. The specific role that the brain area involved in the temporary maintenance of linguistic information and working memory resource allocation exerts in sentence comprehension is still a matter of debate [28,51].

Concerning the analysis conducted with MoCA groupings, again part V presents a correlation with *executive functions* and *orientation*, while part III also correlated with *orientation*. The executive functions refer to complex higher-order functions and are considered to be linked to frontal cortical–subcortical circuits. They are involved in carrying out all the more complex tasks, including attentive supervision and self-monitoring. Instead, the significant relationship between the *orientation area* and the more complex TT subtest could be interpreted as an effect of more severe cognitive impairment: patients who fail in orientation MoCA subtests are those with a severe cognitive impairment and that for this reason are the ones who also fail in the TT complex subtests. On the other hand, it is important to remember that MoCA is a screening test, with very short tests for each cognitive function, and for this reason it does not provide a detailed neuropsychological investigation.

Our results suggest that the existence of an association between auditory–verbal span/auditory working memory and TT performance in right-hemisphere stroke patients. Considering that both right and left hemisphere lesions can compromise short-term and working memory [52,53,54], the effect presented in right hemisphere patients might also be present in left-hemisphere-damaged patients. Hence, our results might support the hypothesis that the TT, and when used with aphasic patients, is probably affected not only by linguistic but also by non-linguistic abilities.

However, further considerations are necessary. Not only the cognitive functions are mutually influenced, but also the neuropsychological tests aimed at assessing one specific function are in some measure influenced by other cognitive performances. For example, the digit span includes a linguistic component and attention underlies all other cognitive functions. Each conclusion must therefore be interpreted considering the numerous factors co-involved in the phenomenon observed. Furthermore, the TT Italian normative data do not need correction for age, while in other languages, such as German, a correction for demographic information is required. This could have influenced the cut-off, which is particularly high in the Italian population, in particular for the assessment of older persons.

From a pragmatic, clinical practice perspective, our results point out other factors that may affect performance at the TT. Evaluating a non-aphasic right hemisphere patient with a TT may thus reveal or confirm impairments in the short term and working memory, while, when administering a TT to an aphasic patient, we must consider that the performance could also be affected by working memory components and attention. The clinical reasoning on the pattern of errors in a TT as well as in other neuropsychological tests, and the observation and interpretation of the patient’s behavior during the test administration, may guide the examiner in the analysis of the verbal comprehension disorder and help to adequately weigh the influence of linguistic and extra-linguistic factors, respectively.

This study presents several limitations. First of all, the sample size is numerically restricted. This did not allow further analyses, such as the evaluation of a possible age effect by a further division into groups. Moreover, to assess the cognitive deficits related to the TT’s performance, we used screening tests, which are not as sensitive as a complete neuropsychological evaluation. Finally, a complete and exhaustive assessment of the language skills of patients was also not performed.

On the other hand, the strengths of this study lie in its prospective nature, as well as in the homogeneity of this cohort, in reference to distance from the acute event.

From the clinical perspective, our results emphasize the importance to investigate verbal short-term memory when assessing and treating verbal comprehension disorders, screening for possible deficits and eventually addressing them with appropriate cognitive rehabilitation treatment. Furthermore, our results also suggest that some caution should be adopted in interpreting TT results in aphasic patients. Indeed, TT errors may not highlight an exclusive problem in linguistic decoding, so the potential effect of other extra-linguistic factors must also be taken into account. This would imply that tests that are less dependent on these highlighted cognitive factors should possibly be used to assess verbal comprehension along with the TT.

In fact, the “Aachener Aphasie Test” evaluation battery [12], which comprises the Token test, includes other tests of lexical and morphosyntactic comprehension, and it is important to consider all of them together to provide a clinical diagnosis. Other tests, included in the classical aphasia evaluation batteries, should also be considered along with the TT to fully investigate verbal comprehension and to obtain a complete profile of the communication skills in aphasic patients. Among them, there are the picture indication tasks (e.g., “Boston Diagnostic Aphasia Examination”, Goodglass and Kaplan, [55]; “Western Aphasia Battery”, Risser et al. [56]; “Esame Neuropsicologico per l’Afasia”, Capasso and Miceli, [57]), as well as a more recent test that investigates both the level of syntactic complexity and the memory–attention load required to perform the task (e.g., “Comprendo”, Cecchetto et al. [58]). Indeed, if our results will be confirmed by subsequent research, we should consider the TT as an integrative and non-exclusive evaluation tool for verbal comprehension in aphasic patients. From this perspective, by only considering the possible extra-linguistic influences observed during TT administration and supplementing the evaluation with lexical and grammatical comprehension tests, the clinician will be able to globally interpret verbal comprehension disorders.

## Figures and Tables

**Table 1:** Demographic and clinical characteristics of the sample (*n* = 37).

Variables	Median [IQR] Count (%)
Age (years)	73.5 ± 13.9
Sex (female)	14 (37.8%)
Education (*years*)	10 [7]
Stroke etiology	
Ischemic	22 (59.5%)
Hemorrhagic	15 (40.5%)
Lesion location	
Subcortical	14 (37.8%)
Lobar	16 (43.2%)
Other	7 (18.9%)
NIHSS score	7.0 ± 4.6

NIHSS = National Institutes of Health Stroke Scale; IQR: interquartile range; %, percentage.

**Table 2:** Neuropsychological Test results of the study sample.

Variables	Mean ± SD Median [IQR]	Range Min, Max
FDS corrected score	5.7 ± 1.1	3.5, 7.9
BDS corrected score	4.0 ± 1.2	1.8, 7.1
MoCA corrected score	19.5 ± 3.8	10.6, 26.6
Visuospatial	2.2 [1.6]	−0.3, 15.9
Attention	4.8 [2.1]	1, 6.2
Language	4.3 [1.7]	1.8, 5.7
Executive	2.3 [1.3]	1.0, 7.9
Memory	1.6 [1.9]	−0.7, 4.8
Orientation	5.0 [1.7]	0.2, 6.1

FDS = Forward Digit Span; BDS = Backward Digit Span; MoCA = Montreal Cognitive Assessment; IQR: interquartile range.

**Table 3:** Partial correlation analyses between Token Test errors and corrected digit span forward and backward scores, adjusted by age.

	Median [IQR]	FDS Corrected Score		BDS Corrected Score	
		r	*p*-Value	r	*p*-Value
TT subpart I	0 [0]	−0.064	0.712	0.181	0.290
TT subpart II	0 [1]	−0.210	0.219	−0.066	0.702
TT subpart III	0 [2]	−0.219	0.200	−0.296	0.080
TT subpart IV	1 [4]	**−0.394**	**0.017**	0.010	0.954
TT subpart V	2 [5]	**−0.408**	**0.013**	**−0.371**	**0.026**

FDS = Forward Digit Span; BDS = Backward Digit Span; TT = Token Test.

**Table 4:** Partial correlations between Token Test errors and Montreal Cognitive Assessment (MoCA) domains, adjusted by age.

	Visuospatial		Attention		Language		Executive Functioning		Memory		Orientation	
	r	*p*-Value	r	*p*-Value	r	*p*-Value	r	*p*-Value	r	*p*-Value	r	*p*-Value
TT sub I	0.234	0.182	0.059	0.741	0.023	0.899	0.007	0.968	−0.082	0.645	−0.141	0.428
TT sub II	0.048	0.788	−0.118	0.508	−0.282	0.106	−0.228	0.195	−0.060	0.738	−0.133	0.454
TT sub III	0.025	0.889	−0.231	0.189	−0.101	0.571	−0.299	0.086	−0.132	0.455	**−0.504**	**0.002**
TT sub IV	0.110	0.535	−0.241	0.169	−0.089	0.617	−0.121	0.494	−0.060	0.738	−0.006	0.975
TT sub V	−0.170	0.337	−0.284	0.104	−0.231	0.188	**−0.468**	**0.007**	−0.032	0.856	**−0.499**	**0.003**

TT = Token Test. Each MoCA domain score is corrected for age, sex, and education.

## Data Availability

The data presented in this study are available on request from the corresponding author.

## Supplementary Materials

The following supporting information can be downloaded at: https://www.mdpi.com/article/10.3390/bs12120494/s1, File S1: Lesion sites, number of subjects and percentage in the sample. Table S1: Token Test (TT) errors (subparts I to V) by age groups. Data are reported as median [interquartile range].
