# Supplementary material for: Extra-Linguistic Cognitive Functions Involved in the Token Test: Results from a Cohort of Non-Aphasic Stroke Patients with Right Hemisphere Lesion
Source: Behav Sci (Basel). 2022 Dec 3;12(12):494. doi: 10.3390/bs12120494 (PMC9774596; doi:10.3390/bs12120494)
Supplement: Supplementary file 1 [file behavsci-12-00494-s001.zip › behavsci-1933871-supplementary.pdf]

Supplementary materials

File S1. Lesion sites, number of subjects and percentage in the sample:

1 (2.7%) frontal+parietal+temporal+basal ganglia

1 (2.7%) frontal+parietal+thalamus+basal ganglia

1 (2.7%) frontal+temporal+thalamus

1 (2.7%) parietal+temporal+insula

1 (2.7%) frontal+temporal+insula

1 (2.7%) temporal+basal ganglia+corona radiata

1 (2.7%) frontal+ temporal+occipital

1 (2.7%) frontal+parietal+thalamus

1 (2.7%) frontal+temporal+thalamus

1 (2.7%) frontal+posterior mesial frontal

1 (2.7%) frontal+basal ganglia

1 (2.7%) parietal+temporal

2 (5.4%) basal ganglia+capsula

2 (5.4%) thalamus+basal ganglia

1 (2.7%) thalamus+pyramidal tracts

1 (2.7%) basal ganglia+internal capsule

1 (2.7%) subcortical-mid-brain

1(2.7%) pons+mid-brain right

1 (2.7%) temporal

3 (8.1%) frontal

2 (5.4%) parietal

6 (16.2%) basal ganglia

1 (2.7%) subtentorial lacunar ischemic stroke

1 (2.7%) brain stem

1 (2.7%) right posteriore limb of the internal capsule

2 (5.4%) cerebellum

Table S1. Token Test (TT) errors (subparts I to V) by age groups. Data are reported as median [interquartile range].

	Age <71 years (N=12)	Age 71-81 years (N=12)	Age>81 years (N=13)
TT subpart I	0 [0]	0 [0]	0 [1]
TT subpart II	0 [0]	0.5 [1]	0 [3]
TT subpart III	0 [0]	0 [0]	2 [2]
TT subpart IV	0 [0]	1.5 [3]	3 [5]
TT subpart V	1 [2]	1 [5]	5 [4]
